# Conference report: Undergraduate family medicine and primary care training in Sub-Saharan Africa: Reflections of the PRIMAFAMED network

**DOI:** 10.4102/phcfm.v9i1.1351

**Published:** 2017-01-30

**Authors:** Innocent Besigye, Robert Mash, Akye Essuman, Maaike Flinkenflögel

**Affiliations:** 1 Department of Family Medicine, Makerere University, Uganda; 2 Division of Family Medicine and Primary Care, Stellenbosch University, South Africa; 3 Family Medicine Unit, Department of Community Health, School of Public Health, University of Ghana, Ghana; 4 Department of Family Medicine and Primary Health Care, Ghent University, Belgium; 5 KIT, The Netherlands

## Abstract

Internationally, there is a move towards strengthening primary healthcare systems and encouraging community-based and socially responsible education. The development of doctors with an interest in primary healthcare and family medicine in the African region should begin during undergraduate training. Over the last few years, attention has been given to the development of postgraduate training in family medicine in the African region, but little attention has been given to undergraduate training. This article reports on the 8th PRIMAFAMED (Primary Care and Family Medicine Education) network meeting held in Nairobi from 21 to 24 May 2016. At this meeting the delegates spent time presenting and discussing the current state of undergraduate training at 18 universities in the region and shared lessons on how to successfully implement undergraduate training. This article reports on the rationale for, information presented, process followed and conclusions reached at the conference.

## Introduction

Sub-Saharan Africa has the highest global burden of disease and the lowest number of health workers per population.^1^ In a resource-constrained environment like this, a focus on primary healthcare (PHC) is most likely to impact on the health of the population and to be cost-effective.^2^ Medical generalists have the competences needed in the multidisciplinary team to build a strong PHC system.^3^ However, in many African countries, PHC is the weakest part of the healthcare system, which often focuses on a limited number of priority conditions that are addressed through fragmented vertical programmes offered by health workers with limited training, skills and support.^2^

Family medicine is the academic discipline that trains doctors and sometimes other health professionals to work as medical generalists. However, in many African countries, the discipline of family medicine is struggling to establish itself in the educational and health systems, often because of the low status of PHC and district health services.^4^ In the African public sector, first contact care is rarely offered by doctors and is most likely to be obtained from a nurse, clinical officer (mid-level clinician) or community health worker.^5^ However, there is growing recognition that family physicians should be part of the multidisciplinary PHC team.^6^ Family physicians are doctors who have completed postgraduate training in order to become expert generalists.^5^ In the African context, the family physician is usually trained to work at the district hospital (sometimes called primary hospital or sub-district hospital) as well as in primary care facilities and community-based services.^5^ Therefore, family physicians are the appropriate physicians to serve in the essential PHC teams.

While many countries in Sub-Saharan Africa have started to offer family medicine training at a postgraduate level, there is also a need for exposure to PHC at the undergraduate level. In the undergraduate curriculum, the discipline of family medicine does not contribute knowledge of a new pathophysiological system, but exposes students to a different context of care that is closer to the community and the burden of disease.^7^ Training in this context relates to the health problems that the majority of people experience and increases the social accountability of medical schools.^8^ Family medicine has holistic care as a core principle and emphasises the use of patient-centred consultation skills.^9^ Family medicine embraces the core principles of PHC, such as accessibility, continuity of care, co-ordination of care and comprehensiveness.^10^ As medical schools aspire to train basic doctors responsive to the needs of the community they serve, these PHC principles are essential. This implies that family medicine should be at the heart of the curriculum and not added as an after-thought.

Over the last few decades, there has been a global shift towards community-based education.^8^ Medical schools have recognised that teaching doctors in a tertiary hospital setting has substantial limitations as they do not represent the common reasons for encounter and conditions seen in the majority of the population.^8^ Social accountability of medical schools requires them to prepare doctors better for the needs of society and to foster health systems that promote universal coverage and equity.^8^ The Lancet Commission on the future of health professionals education criticises medical schools for curricula that have a ‘narrow technical focus without broader contextual understanding; episodic encounters rather than continuous care; predominant hospital orientation at the expense of primary care’.^8^ The result may be a newly qualified doctor whose competencies are mismatched with the patient and population health needs.

The PRIMAFAMED network is an educational and research network of departments of family medicine in Sub-Saharan Africa.^11^ Historically, it has focused on supporting the development of postgraduate training in family medicine, but at the 2016 meeting in Nairobi, Kenya, the network devoted time to evaluate and reflect on undergraduate education in the region.

## Methods

All participating institutions in the 8th PRIMAFAMED meeting conducted from 21 to 24 May 2016 were requested to prepare a poster for presentation detailing the following: presence or absence of family medicine and PHC in the undergraduate curriculum, what teaching is delivered by family medicine in the curriculum on campus, what clinical training is provided at family medicine teaching sites, duration of exposure, average number of students, number of staff, types of teaching and geographical spread, how assessment is done, lessons learnt and future plans. Delegates whose institutions did not yet embrace family medicine training in undergraduate medical education were requested to share any plans for the future verbally with the group.

A 3-hour poster session was facilitated. Posters were randomly grouped into three clusters. Participants, if they were not presenters, were also randomly divided into three groups, and each group was assigned a cluster of posters to view and discuss. Each presenter was requested to summarise the salient points from the poster to the group in 5 minutes, and the groups were then able to ask questions for another 5 minutes. Each group was moderated by the appointed chairperson.

After the walk-about poster session, each group met in a breakaway room to discuss the following question: what lessons can we learn on how to successfully implement undergraduate family medicine and PHC training in Sub-Saharan Africa? Finally, the three groups convened in a plenary session where each group made a 10-minute oral presentation followed by a brief discussion.

## Results

Eighteen African institutions presented posters on their undergraduate education in family medicine and PHC. There was significant heterogeneity in approaches to undergraduate family medicine training between the different institutions.

In Southern Africa, undergraduate family medicine education was well established in South Africa and had been introduced in Botswana and Malawi, but not in Zimbabwe, Namibia or Mozambique (Lurio University).

In East Africa, undergraduate education was present in some Kenyan and Ugandan universities. In Kenya, undergraduate education was present at Moi University, but not at Maseno, Aga Khan, Kenyatta or Nairobi. In Uganda, undergraduate education was present at Makerere University, Mbarara University of Science and Technology and Kampala International University. In Rwanda, the family physicians on faculty contributed to an undergraduate social and community medicine programme that included population health, health systems, social medicine, professionalism and communication. Tanzania had no undergraduate education in family medicine.

In West Africa, undergraduate family medicine education was established in Nigeria and Ghana. The University of Calabar and Lagos University had well-established programmes. Ghana started an undergraduate programme at the University of Ghana in 2008, but it is yet to be well established. Four other universities in Ghana are yet to start the programme.

In most universities without a formal presence of family medicine in the undergraduate curriculum, the family medicine department contributes indirectly in areas such as community health and communication skills.

Table 1 summarises the undergraduate programmes at the institutions that have implemented training.

**TABLE 1:** Description of undergraduate programmes in Sub-Saharan Africa.

Country	University	Year when programme started	Duration of exposure (weeks)	Description	Learning outcomes	Assessment
Nigeria	University of Lagos	2008	6	Lectures and theoretical teaching sessions provided at family medicine unit located at the College of Medicine. Clinical training takes place in 50 general practices attached to the College, but located in the community.	Focus on theories, concepts and principles of family medicine in line with the competences of family physicians as communicator, collaborator, manager, scholar, medical generalist and health advocate.	Progressive and final written exam and OSCE.
Nigeria	University of Calabar	1983	46	30 weeks shared with surgery, medicine, pharmacology and laboratory medicine and 16 weeks shared with community medicine. Clinical rotations done in clinics with private practitioners supervising the learning.	Focus on learner acquiring knowledge, skills and attitudes appropriate to primary care practice in both urban and rural settings.	Logbook, written assignment, MCQs, OSCE.
Ghana	University of Ghana	2008	2.5	One-and-half weeks of didactic lectures on ‘Principles of family medicine’ in 4th year. Four days of clinical training on ‘The practice of family medicine’ in 6th year.	Focus on theories, concepts and practice of family medicine. The settings for practice include both public and private primary care facilities.	Group assignments for formative assessment. No final examinations currently.
Uganda	Kampala International University		10	Ten weeks of family medicine clerkship in the 5th year of study. Clerkship involves 8 weeks of didactic lectures followed by 2 weeks of clinical training within the teaching hospital.	Focus on understanding family medicine, its principles and practice as well as COPC.	Individual student portfolios as continuous assessment and end-of-year examination.
Uganda	Makerere University	2011	4	Four-week clinical rotation in the 4th year of study. Exposure to various primary care settings with general practitioners as clinical supervisors and instructors with the help of a student learning guide.	Focus on introduction to FM, principles of family medicine, the consultation, patient-centred care, biopsychosocial model and family in health and disease.	Both clinical (OSCE) and written examination.
Kenya	Moi University	2015	-	Family health module worth 3 credits via overviews, tutorials and a logbook in 6th year.	Chronic disease management and palliative care.	Written examination.
Rwanda	University of Rwanda	2011	-	Year 1 two (both 10 credits) theoretical modules; year 3 combined theoretical and practical longitudinal (20 credits) module; year 4 practical reflective (15 credits, 4 weeks) module.	Population health, health systems, social medicine, communica-tion, professionalism. The ‘desired Rwandan medical doctor framework’ is used as key (patient-centred and community-oriented care provider, communicator, collaborator, health advocate, manager, scholar and professional).	Formative: assessment of individual and group work. Summative: assessment of behaviour in the field and presentations, written (MCQ/ open questions).
Malawi	University of Malawi	2011	6	Six-week rotation in the family medicine block in the 4th year.	Focus on family medicine as a career, biopsychosocial model, approach to primary care symptoms, chronic care, quality improvement, leadership geriatrics and palliative care.	Log book, written exam and OSCE.
Botswana	University of Botswana	2009	22	FM teaches consultation, communication and clinical skills in years 1, 2, 3 and 5. In years 3 and 5, there are clinical rotations in which students are taught management of patients with common problems in community clinics and hospital settings. They are also introduced to COPC.	Focus on a holistic patient-centred approach in caring for patients, social determinants of disease and an evidence-based approach using available resources.	Assessment: formative and summative: case presentations, problem-based learning assessments, portfolios.
South Africa	University of Kwa-Zulu Natal	2000	33	Three rotations with public health in 2nd, 3rd and 4th years that focus on COPC (14 weeks). Three rotations in integrated primary care at clinics and district hospitals in 4th, 5th and 6th years (19 weeks).	Focus on the development of professional attributes: clinician in primary care, professional, communicator, scholar, collaborator, manager, health advocate.	Assignments, presentations, posters. OSCE, MCQ, clinical cases, portfolio.
South Africa	Stellenbosch	1998	13	Three rotations in early (4-weeks), middle (4-weeks) and late phases (5-weeks). Exposure to primary care, district hospitals, urban and rural settings. Option of Rural Clinical School in final year with traditional rotations or longitudinal clerkship at district hospital. Rotations shared with community health and rehabilitation.	Focus on common reasons for encounter, holistic and patient-centred consultations, common conditions in primary care and district hospital, district health system, clinical governance, teamwork, resilience, ethics, evidence-based medicine, health promotion and disease prevention.	Final assessment based on class mark and OSCE.
South Africa	University of Pretoria		-	Family medicine training is done throughout all the 6 years of undergraduate medical training.	Focus on integration of hospital, clinic and home care using the COPC approach.	Weekly reflective reports, log book and OSCE.
South Africa	University of Cape Town		12 weeks	Twelve weeks of clinical rotation in 4th and 6th years (8 weeks shared with public health and 4 weeks shared with palliative care respectively).	Focus on communication, community health and clinical skills for primary care.	Logbook, written theory examination and OSCE.

OSCE, objective structured clinical examination; MCQ, multiple choice questions; FM, family medicine; COPC, community-orientated primary care.

## Lessons learnt about how to successfully implement undergraduate training in family medicine and primary health care in Africa

The contribution of family medicine to undergraduate training in African medical schools is still small and mostly limited to the final clinical years. There is a need to advocate for it to be included in the curriculum at an earlier stage and for a higher number of credits. In the absence of family physicians as part of the faculty, such advocacy initially needs to come from champions in other disciplines. Advocacy is easier in a context in which government policy prioritises PHC and acknowledges the role for the doctor in primary care. There is also a need to synthesise, create and articulate an evidence base for the value of family medicine in the undergraduate curriculum. Medical schools often start with a small exposure to family medicine, and positive feedback from these students is essential to confirm the value of such training.

In some African countries such as Namibia or Botswana, new medical schools have recently been established. In this context, it is often easier to incorporate family medicine and primary care into the curriculum when it is being initially designed and developed. It is much harder to introduce family medicine into a curriculum that is already established, as this requires other specialist disciplines to reduce their footprint. Curriculum reviews that happen once every 5–10 years also provide an opportunity to advocate for the inclusion of family medicine. Many curricula already include community-based education through a department of community health. Collaboration with departments of community health to combine population and primary care perspectives within the district health services is an effective possibility.

Medical schools are most often established in the context of national- or tertiary-level referral hospitals. Therefore, although the academic departments of family medicine are often administratively located in or alongside the tertiary hospital, it is of utmost importance to teach the students in primary care or the district health services in order for them to understand family medicine and primary care in the relevant context. It is difficult to show the value of family medicine when clinical training is conducted within a tertiary hospital. Ideally, the clinical training should provide continuity with the same community and primary care facilities. In a few universities, such as Stellenbosch in South Africa, medical students are now offered longitudinal clerkships and can spend the whole of their final year in a district hospital setting.^12^ The Lancet Commission also promotes the concept of academic systems that network hospitals and primary care facilties.^8^

Once an opening for family medicine is created in the undergraduate curriculum, one of the early challenges is finding sufficient suitable clinical trainers and to develop sustainability. Having more family physicians trained at a postgraduate level and employed as clinical role models in the district health services is critical. Being exposed to family medicine at an undergraduate level is also one way of increasing interest in postgraduate training. Another strategy is to identify motivated generalist doctors without postgraduate training who can be re-orientated and up-skilled, sometimes through short courses or Diplomas in Family Medicine.^13^ A less satisfactory solution that is currently used is to try and co-opt hospital specialists to train family medicine. Involving the family medicine postgraduate registrars in the undergraduate family medicine training is both beneficial for the undergraduate students who learn from their older colleagues and can see them as role models and for the registrars as they grow in being an educator and mentor.

There is a need to create consensus on the contribution that family medicine can make to the curriculum while at the same time remaining open to innovation. At the moment, there is huge variation in the contribution of family medicine at different medical schools in Africa because of different contextual issues and different understandings of what family medicine means in African health systems. For example, some programmes emphasise ambulatory primary care, while others focus on care in rural district hospitals and others on community-based PHC teams.

## Conclusion

Despite the need for well-trained generalist doctors in the African context to address the burden of disease in PHC and district health services, there is limited space for family medicine and PHC education in most undergraduate curricula. The discipline of family medicine needs to advocate more strongly for inclusion. As departments of family medicine, we need to strengthen collaboration and ensure that such training occurs in community-based settings, to look at innovative strategies to increase the pool of generalist clinical trainers and to articulate models of family medicine that appropriately address the burden of disease and the needs of the community in each country.
